# Structure and function of a silicic acid channel Lsi1

**DOI:** 10.3389/fpls.2022.982068

**Published:** 2022-09-12

**Authors:** Yasunori Saitoh, Michihiro Suga

**Affiliations:** ^1^Research Institute for Interdisciplinary Science, Okayama University, Okayama, Japan; ^2^Graduate School of Natural Science and Technology, Okayama University, Okayama, Japan

**Keywords:** silicon, aquaporin, NIP, rice, crystal structure, substrate selectivity, channel, transporter

## Abstract

Silicon is a beneficial element for plant growth and production, especially in rice. Plant roots take up silicon in the form of silicic acid. Silicic acid channels, which belong to the NIP subfamily of aquaporins, are responsible for silicic acid uptake. Accumulated experimental results have deepened our understanding of the silicic acid channel for its uptake mechanism, physiological function, localization, and other aspects. However, how the silicic acid channel efficiently and selectively permeates silicic acid remains to be elucidated. Recently reported crystal structures of the silicic acid channel enabled us to discuss the mechanism of silicic acid uptake by plant roots at an atomic level. In this mini-review, we focus on the crystal structures of the silicic acid channel and provide a detailed description of the structural determinants of silicic acid permeation and its transport mechanism, which are crucial for the rational creation of secure and sustainable crops.

## Introduction

Plants absorb minerals from the soil through their roots. The type and amount of absorbed minerals vary among plant species, and such differences are associated with the physiological diversity of plants. Numerous studies have been conducted on the usefulness of silicon taken up by plants, beginning with Onodera’s work suggesting that silicon confers resistance for rice blast (Onodera, 1917). It is generally accepted that silicon is not an essential but beneficial element for most plant species (Yamaji and Ma, 2021). Silicon is dissolved in soil solution in the form of silicic acid, which plants absorb through their roots (Takahashi and Hino, 1978). Silicon content in plants varies by two orders of magnitude ranging from 0.1 to 10%. This indicates that the degree to which silicon is utilized varies widely among plant species (Ma and Takahashi, 2002). Among plants, Poaceae, Cyperaceae, Equisetaceae, and some ferns show markedly high silicon content (Ma and Takahashi, 2002; Hodson et al., 2005; Trembath-Reichert et al., 2015). Rice shows an extremely high silicon content, containing silicon in more than 10% of its dry weight (Tamai and Ma, 2003). The amount of silicon is higher than that of the essential plant elements nitrogen, phosphorus, and potassium (Epstein, 1994; Datnoff et al., 1997; Ma et al., 2001). Rice absorbs silicic acid from the soil through its roots, transport it into the xylem vessel, distributes it to various tissues, and eventually deposits it in the form of silica, SiO_2_, in the cell walls and throughout the epidermal cells of leaves, stems, and husk, thereby making the plant body more robust (Ma and Yamaji, 2015; Wang et al., 2017). The silica deposition provides rice plants with various benefits, such as increased epidermal cell hardness, reduced cuticular transpiration, improved light-receiving posture, leading to biotic resistance to funguses, pathogens, pathogenic viruses, and insect pests, abiotic resistance to water loss and lodging, and increased photosynthetic efficiency (Ma, 2004; Andama et al., 2020). The various benefits of silicic acid uptake dramatically improve the growth and productivity of rice.

**Figure 1:**
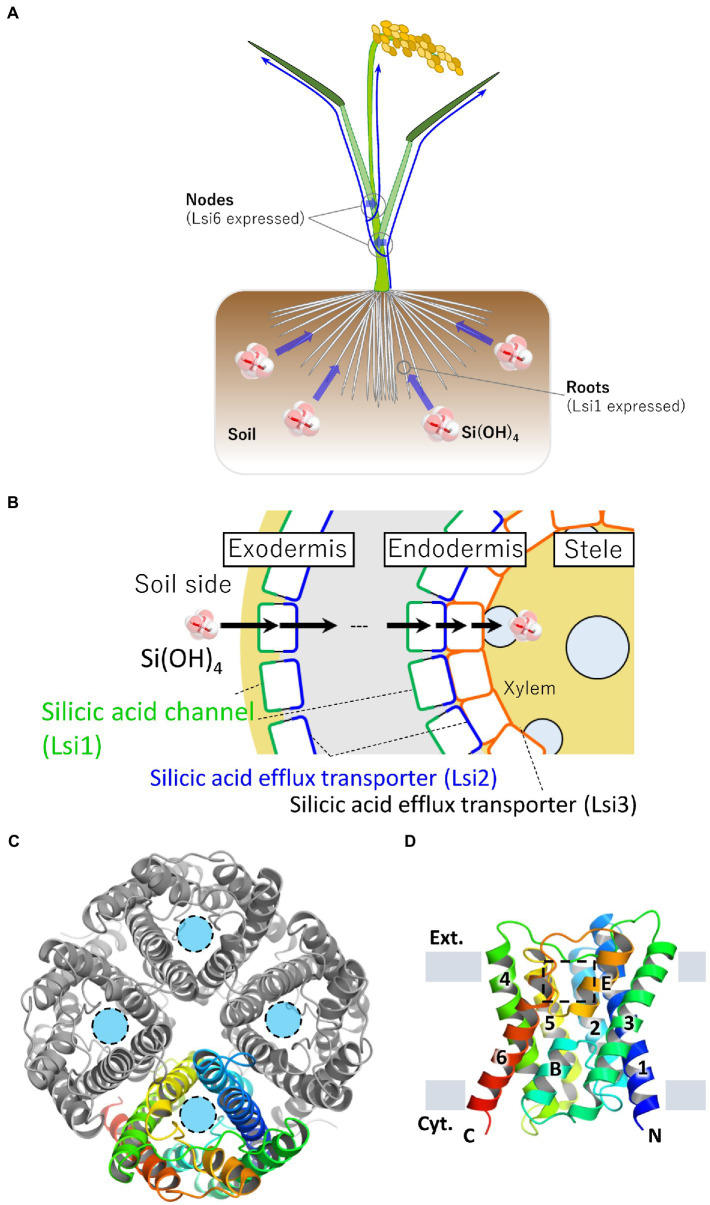
Localization of silicic acid transporters in rice and crystal structure of Lsi1. **(A)** Schematic representation of silicic acid absorption and distribution in rice plants. Rice roots take up silicic acid in the soil. Blue arrows indicate silicic acid migration. Silicic acid channel Lsi1 is mainly localized in the roots, and Lsi6 is localized in the nodes. Lsi1 and Lsi6 are involved in silicic acid absorption and distribution, respectively. **(B)** Schematic cross-section of a rice root. Lsi1, shown in the green line, is localized on the distal side of the cell membrane in the exodermis and endodermis. A silicic acid efflux transporter Lsi2 is shown in the blue line. Lsi2 is localized on the proximal side of the cell membrane in the exodermis and endodermis. Lsi3, shown in the orange line, is localized on the cell membrane in the pericycle. **(C)** The overall structure of the Lsi1 tetramer is viewed from the **FIGURE 1 (Continued)**extracellular side (PDB ID: 7CJS). One protomer is shown in a rainbow, while the others are shown in gray. Light blue circles indicate substrate permeation pathways. **(D)** Side view of the monomeric Lsi1. Numbers indicate TM1~6; B and E indicate short helix B and E, respectively. The grey bars indicate the membrane boundaries. A square in a dashed line indicates the SF region. Panels **(C,D)** are reproductions from Saitoh et al. (2021).

A rice mutant low silicon rice 1 (lsi1) has a significantly reduced silicon uptake capacity compared to the wild type, and the yield of mutant rice is lesser than the wild type due to the reduction in the uptake of silicon (Ma et al., 2002, 2006). The gene responsible for lsi1 mutant, *Lsi1*, was identified as a silicic acid channel gene. It belongs to the Nodulin 26-like intrinsic proteins (NIP) subfamily of the major intrinsic proteins (MIP) family to which aquaporins (AQPs) belong (Sakurai et al., 2005; Ma et al., 2006). Rice has two genes of silicic acid channels, *Lsi1* and *Lsi6*. *Oryza sativa* (Os)Lsi1 is localized mainly in roots and involved in silicic acid loading, whereas OsLsi6 is localized mainly in nodes and is involved in silicic acid unloading from the xylem vessels (Yamaji et al., 2008, 2015; Figure 1A). Cellular localization of Lsi1 orthologs in roots varies in plant species (Ma and Yamaji, 2015). In rice roots, OsLsi1 is localized on the distal side of the cell membrane in the exodermal and endodermal cell layers separated by the Casparian stripes (Yamaji and Ma, 2007). Two genes of silicic acid transporters in rice, *Lsi2* and *Lsi3*, have also been identified (Ma et al., 2007; Yamaji et al., 2015). OsLsi2 and OsLsi3 have no sequence similarity with the silicic acid channels and actively transport silicic acid. OsLsi2 is localized on the proximal side of the cell membranes in the exodermis and endodermis with the opposite polarity to OsLsi1 and functions as a silicic acid efflux transporter. Thus, silicic acid from the soil is efficiently transported into the xylem vessels by the cooperative action of two completely different membrane proteins, OsLsi1 (passive transport) and OsLsi2 (active transport; Ma et al., 2007; Sakurai et al., 2015; Konishi et al., 2022; Figure 1B). OsLsi3 is localized to the pericycle in the roots without polarity and is involved in the xylem loading of silicon (Huang et al., 2022). Therefore, rice has an efficient transport system for the uptake, translocation, and distribution of silicon which is enabled by these silicon channels and transporters. Since the identification of the *Lsi1* gene in rice, several silicic acid channel genes have been identified and characterized in various angiosperms (Ma and Yamaji, 2015). In Equisetaceae, which requires silicon as an essential element, silicic acid channels (EaNIP3s) and silicic acid efflux transporters (EaLsi2s) with low sequence homology to those of angiosperm have also been identified (Ea for *Equisetum arvense*, Grégoire et al., 2012; Vivancos et al., 2016). This mini-review provides an overview of the function and recently elucidated structures of the silicic acid channel Lsi1.

### The function of silicic acid channel Lsi1

Identification of the *Lsi1* gene revealed that Lsi1 belongs to the NIP subfamily of the AQP family (Ma et al., 2006). This finding has further accelerated the study of OsLsi1 based on the accumulated structural and functional insights from the extensive AQP research. In the substrate pathway (channel), its narrowest part mainly determines substrate selectivity of the AQP family, the so-called selectivity filter (SF), consisting of four amino acid residues. Animal and bacterial AQPs are roughly classified into two groups with different SFs based on amino acid sequence homology; AQP, which selectively permeates water only, and aquaglyceroporin, which selectively permeates glycerol as well as water (Gonen and Walz, 2006). On the other hand, plant AQPs are significantly more diverse compared to that of animals and bacteria and are classified into five subfamilies; the plasma membrane intrinsic proteins (PIP) subfamily, the tonoplast intrinsic proteins (TIP) subfamily, the small basic intrinsic proteins (SIP) subfamily, NIP subfamily, and the X intrinsic proteins (XIP) subfamily, based on amino acid sequence (Abascal et al., 2014). The subfamilies are further classified into several subgroups according to the type of amino acid residues in the SF (Johanson and Gustavsson, 2002; Wallace et al., 2002; Wallace and Roberts, 2004, 2005; Rougé and Barre, 2008). Sequence analysis and mutagenesis experiments have shown that Lsi1 belongs to the NIP-III subgroup with a distinctive SF consisting of small amino acid residues (G-S-G-R), which are essential for silicic acid permeation (Bansal and Sankararamakrishnan, 2007; Mitani et al., 2008; Rougé and Barre, 2008; Mitani-Ueno et al., 2011; Hayes et al., 2013; Vatansever et al., 2017). However, SF replacing experiment suggested that other parts of the SF are also crucial for silicic acid permeability (Mitani-Ueno et al., 2011). For instance, spacing between the two Asn-Pro-Ala (NPA) motifs has been proposed to be crucial for silicic acid permeability in tomato and poplar Lsi1 (Deshmukh et al., 2015; Sun et al., 2020). Substrate transport in Lsi1 is passive, driven by concentration gradients, and its transport is bidirectional (Mitani et al., 2008). Lsi1 also plays a significant role in the uptake of boric acid to utilize it as a source of essential element boron (Mitani et al., 2008; Mitani-Ueno et al., 2011; Shao et al., 2018). Lsi1 is also involved in the transport of arsenite. Therefore it is involved in the uptake and efflux of arsenite in rice when grown in irrigation water and upland cultivation, respectively (Ma et al., 2008; Mitani et al., 2008; Schnurbusch et al., 2010; Zhao et al., 2010). Lsi1 has high selectivity for silicic acid over glycerol or boric acid, smaller molecules than silicic acid with similar properties. How Lsi1 efficiently conducts silicic acid with high selectivity has been a mystery for a long time until its structure was clarified (Ma et al., 2006; Mitani et al., 2008; Pommerrenig et al., 2015; Luang and Hrmova, 2017; Roberts and Routray, 2017).

### Structures of rice Lsi1

**Figure 2:**
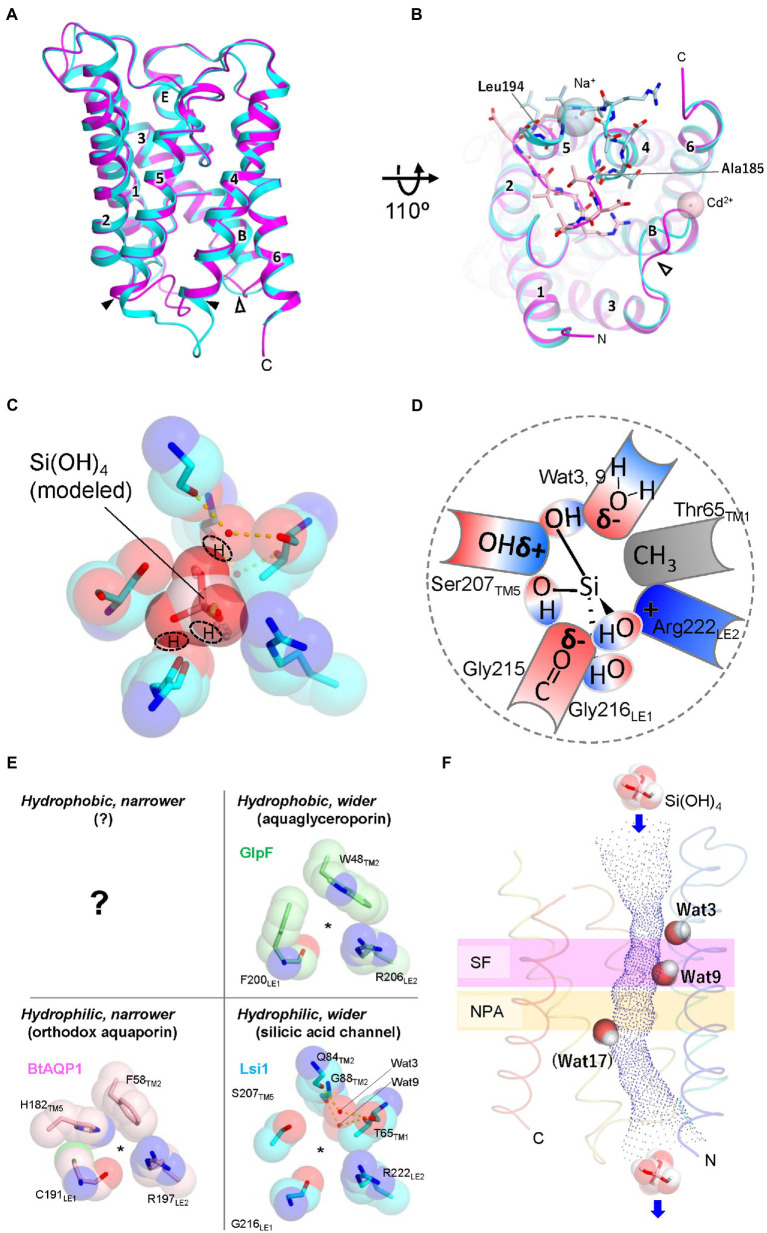
Structures of Lsi1 and silicic acid permeation model in SF. **(A)** Superposition of the Lsi1 structures in the open state (PDB 7CJS, cyan) and closed state (PDB 7NL4, magenta). Numbers indicate TM1~6; Alphabets indicate Short helices B and E. The solid and open arrowheads indicate loop D and loop B, respectively. **(B)** The view from the Intracellular side. Amino acid residues of loop D are shown as stick representations in light blue (open state) and light pink (closed state). Spheres indicate Na^+^ ion (open state, light blue), and Cd^2+^ ion (closed state, light pink). In the closed state, **FIGURE 2 (Continued)**loop D blocks the channel of Lsi1. Loop B structure in the closed state differs from the open state structure probably due to the crystal packing, Cd^2+^ ion, and interactions with loop D. **(C)** The SF of Lsi1 is viewed from the extracellular side (PDB ID: 7CJS). **(C)** Silicic acid molecule was manually placed based on the oxygen atoms of the water molecules in the crystal structure and examined by the QM/MM calculation. The predicted OH groups of silicic acid are indicated. **(D)** Schematic diagram of silicic acid passing through the SF. The OH groups of silicic acid pass through three crevices in the SF; the first OH group passes between Arg222 and the carbonyl ladder, the second one passes between the carbonyl ladder and Ser207, and the third one passes between Ser207 and a polar face made by waters (Wat3, Wat9). δ− and δ+ indicate bond polarity. **(E)** Classification of SFs by pore size and hydrophilicity. BtAQP1 (PDB 1J4N), GlpF (PDB 1FX8), and Lsi1 (PDB 7CJS). A question mark indicates that no such SFs have yet been identified. **(F)** Unique water molecules (Wat3, Wat9, and Wat17) in the Lsi1 channel limit silicic acid permeation. Channel profile along the Lsi1 pore calculated using the program HOLE2 is also shown. The SF and NPA motifs regions are colored in plum and khaki, respectively. Wat17 was observed only in the MD simulation. Panels C and E are reproductions from Saitoh et al. (2021).

Recently, structures of Lsi1 from rice (OsLsi1/OsNIP2;1) have been reported by two research groups (Saitoh et al., 2021; van den Berg et al., 2021). Both X-ray structures were different in resolutions and channel states. Saitoh et al. reported an open state structure at a resolution of 1.8 Å. In contrast, van den Berg et al. reported a closed state structure at a resolution of 3.0 Å. Since the physiological state of Lsi1 is in an open conformation and the high-resolution structure showed hydrogen bonding interactions of Lsi1, including many water molecules bounds to the channel, the 1.8-Å resolution structure is helpful for further analysis. The latter structure is helpful in discussing the channel gating mechanism. The structures of OsLsi1 showed similar folding to other AQP family proteins and formed a homotetramer (Figure 1C). Each monomer contains six transmembrane helices (TM1-TM6), five connecting loops (loop A-loop E), and two half helices (HB and HE) with N and C-terminus located on the cytoplasmic side of the membrane (Figure 1D). The channel of Lsi1 exists in each protomer’s center, similar to the other AQPs (Figure 1C). However, the transmembrane helical orientations are different from the other AQPs (Saitoh et al., 2021). The orientation differences in transmembrane helices should also affect the shape and nature of the Lsi1 channel. The Lsi1 channel is wider than the AQP (*Bos taurus* (Bt)AQP1, Sui et al., 2001) and aquaglyceroporin (GlpF, Fu et al., 2000). The narrowest part of the Lsi1 channel is the SF in the open state (Saitoh et al., 2021). In contrast, loop D on the cytoplasmic side blocks the channel in the closed state, while the SF is similar to the open state (van den Berg et al., 2021; Figures 2A,B). The SF of AQPs and aquaglyceroporins consists of four or three amino acid residues, including Arg and aromatic residues. However, the SF of Lsi1 consists of five amino acid residues and two unique water molecules (Figures 2C,D). Four of the five amino acid residues (G-S-G-R) are consistent with the previous prediction by comparing AQPs structures and amino acid sequence. The fifth amino acid residue, Thr65, extends from TM1 and binds two unique water molecules, Wat3 and Wat9. These two water molecules with the oxygen atom facing the channel lumen are located on the opposite side of the carbonyl ladder, which is well conserved in AQP structures. The SF of Lsi1 is broader and more hydrophilic than AQPs and GlpF (Figure 2E). One exception is the SF of human aquaglyceroporin, hAQP10, which is similar to Lsi1 and has been shown to pass through silicic acid, suggesting a convergent evolution (Garneau et al., 2015; Gotfryd et al., 2018; Saitoh et al., 2021).

### The regions responsible for a silicic acid permeation

Steered molecular dynamics (MD) force profiles show that the SF requires the most energy to pass silicic acid through the channel, indicating that the SF is most important for silicic acid selectivity (van den Berg et al., 2021). Mutations into the SF, both in loss of function and gain of function, have demonstrated the importance of SF in silicic acid permeation and selectivity (Mitani-Ueno et al., 2011; Hayes et al., 2013; Saitoh et al., 2021). However, the Lsi1-like replacement in the SF of GlpF showed silicic acid permeability and decreased glycerol permeability (Saitoh et al., 2021), whereas the equivalent replacement into the boric acid channel in *Arabidopsis thaliana*, AtNIP5;1, did not confer silicic acid permeability (Mitani-Ueno et al., 2011). These experiments indicate that regions other than SF also involve silicic acid permeability. Deshmukh et al. presented that a length of 108 amino acids between two NPA motifs in Lsi1 is essential for silicic acid permeability based on functional analysis results using deletion or insertion mutations in loop C and amino acid sequence comparison (Deshmukh et al., 2015). However, the GlpF mutant, whose length between NPA motifs is not 108 amino acids, shows silicic acid permeability by replacing SF with that of Lsi1. Since structural analysis shows that loop C and SF have extensively hydrogen-bonded interactions, including Gly155 to Arg222, Thr157 to Thr223, and Val160 to Ile213, stabilization by such interactions would be necessary for silicic acid permeability rather than the length between NPAs (Saitoh et al., 2021; van den Berg et al., 2021). A plant ammonia-permeable AQP, AtTIP2;1, is another example that loop C affects SF and channel permeability (Kirscht et al., 2016). Another residue affecting silicic acid permeability and selectivity is Thr181 of TM4, located on the channel lumen. The position of Thr181 is pseudo-*c*2-symmetrically related to Thr65 of SF (Saitoh et al., 2021). The functional analysis based on the Lsi1 structure demonstrates several factors for silicic acid permeation other than SF.

### A mechanism to avoid proton leak

Maintaining proton gradients across cell membranes is essential for the bioenergetics of any living cell, as the resulting proton-motive force drives numerous transport processes, membrane fusion, and ATP synthesis. One of the essential properties of AQPs is preventing proton leakage while allowing fast substrate permeation. Protons in the bulk water can move quickly through hydrogen-bonded water molecules *via* the Grotthuss mechanism by exchanging a covalent bond between H and O with the neighboring water molecules (Cukierman, 2006). Why this does not happen in AQP is an exciting question, and many researchers have proposed various mechanisms (Murata et al., 2000; de Groot and Grubmüller, 2001, 2005; Tajkhorshid et al., 2002; Burykin and Warshel, 2003; de Groot et al., 2003; Chakrabarti et al., 2004). The prevailing view is that positive electrostatic potential created by NPA motifs and disrupted hydrogen bonds between water molecules in SF disable proton transfer *via* the Grotthuss mechanism through single-file water molecules in the channel (Beitz et al., 2006; Wu et al., 2009; Kosinska Eriksson et al., 2013). Structural analysis of OsLsi1 at 1.8 Å resolution revealed that, unlike other AQPs and aquaglyceroporins, there are many water molecules in the Lsi1 channel and that they are no longer single-file in SF. The vast and hydrophilic SF of Lsi1 likely brought this unique arrangement. The SF of Lsi1 seems to have its own proton exclusion mechanism distinct from AQPs. Saitoh et al. argue that the unique water molecule (Wat9) in SF is hydrogen-bonded to the intracellular side single-file water molecules but can only act as a hydrogen bond acceptor, which may inhibit proton jumping *via* the Grotthuss mechanism (Saitoh et al., 2021). Further MD simulation and higher resolution structure analysis should validate the mechanism preventing proton transfer.

### The silicic acid permeation mechanism

For further understanding of the silicic acid permeation in Lsi1, the silicic acid-bound Lsi1 structure is inevitable. Saitoh et al. tried soaking crystals in a solution containing silicic acid or germanic acid, a silicic acid analog. However, they failed to detect anomalous X-ray scattering signals derived from silicic or germanic acid, possibly due to their low solubility or weak affinity with Lsi1. Saitoh et al. used coordinates of oxygen atoms of the water molecule in the open state structure as a guide and manually placed the silicic acid molecule (Figure 2C). Examination by quantum mechanical/molecular mechanical (QM/MM) calculations with the model indicated putative amino acid residues interacting with silicic acid and the mode of interactions. As silicic acid is tetrahedral with OH groups at their vertices, the projected view from any vertices is a triangle. The SF of Lsi1 has three crevices where the triangle fits well. Therefore, it is reasonable if OH groups of silicic acid pass through the crevices while forming a hydrogen bond with the SF (Figures 2C,D). MD simulations have shown that silicic acid passes through the channel of Lsi1 (Saitoh et al., 2021; van den Berg et al., 2021). MD simulation also revealed three bottlenecks for silicic acid permeation in the Lsi1 channel (Saitoh et al., 2021). Each bottleneck has a stably occupying water molecule. Two water molecules (Wat3 and Wat9) formed bottlenecks in the SF by occupying positions consistent with the crystal structure. However, the other water molecule (Wat17) hydrogen-bonded to Thr181 occupied a position where no water was identified in the crystal structure, forming a bottleneck on the cytoplasmic side (Figure 2F). The difference between the crystal structure and theoretical calculations regarding Wat17 may arise from the protein environment (lipid membrane vs. micelle; Fujiyoshi, 2011). Wat3 and Wat17 are dislodged when silicic acid passes through these bottlenecks, but Wat9 remains in the SF during silicic acid permeation and acts as a hydrogen bond acceptor. These water molecules seem to strictly constrain the orientation of a silicic acid passing through the channel. The water molecules likely stabilize the bottlenecks due to the strong hidrogen bond interactions, but further theoretical analysis is necessary. Thr65 and Thr181 are identified to be important for silicic acid permeation. However, they are not conserved in the Leguminosae among angiosperms. The difference may be related to the substrate specificity of the channel. The only known regulation of Lsi1 channel activity is that it is reduced by adding HgCl_2_ (Mitani et al., 2008). As noted, van den Berg et al. reported the structure of Lsi1 with loop D closing the channel on the cytoplasmic side (van den Berg et al., 2021). *Spinacia oleracea* PIP2;1, a member of the PIP subfamily, has also been reported to be involved in channel gating by cytoplasmic loop D, but its gating mechanism seems to be different from that of Lsi1 (Törnroth-Horsefield et al., 2006). How the loop D changes its structure during open to close state and its relevance to the HgCl_2_ inhibition, and its physiological significance, remain to be elucidated.

## Perspectives

Research on silicic acid channels has progressed very rapidly by intermingling with well-studied research on AQPs. Recently analyzed structures of the silicic acid channel OsLsi1 provide structural insights into the silicic acid permeation mechanism of Lsi1. The structures would boost further studies on the silicic acid permeation mechanism, proton exclusion mechanism, the regulatory mechanism of silicic acid channel activity, and the application of silicic acid channels to modify substrate selectivity. In particular, since high concentrations of arsenic accumulate in rice in areas contaminated with arsenic (Hassan et al., 2017), it would be helpful to develop rice varieties that are impervious to arsenic, or conversely, plants that take up a high level of arsenic and clean up the soil (Bienert et al., 2008). As the example of Lsi1 shows, plant AQPs are highly diversified, and it is of great interest and importance to understand the mechanisms of substrate selectivity. Structural analysis of the silicic acid channels of the Equisetaceae (Grégoire et al., 2012), which seems to have a different SF from that of Poaceae, is necessary. New knowledge obtained from the structural analysis of Lsi1 may help to clarify the origin of plant NIPs which is controversial currently (Zardoya et al., 2002; Gustavsson et al., 2005; Bhattacharjee et al., 2008; Danielson and Johanson, 2008, 2010; Anderberg et al., 2011, 2012; Abascal et al., 2014; Perez Di Giorgio et al., 2014; Finn and Cerdà, 2015; Pommerrenig et al., 2020). Structural studies of the silicic acid efflux transporters, Lsi2 and Lsi3, should be conducted to elucidate the mechanism of silicic acid uptake in plants at the atomic level. Research on the silica deposition mechanism is also needed (Shivaraj et al., 2021). Silicon is the only element that makes plants more robust without excess toxicity. The silicon transporters, Lsi proteins, are acquired only by certain plant species and are highly specialized. Studying its structure is essential for understanding the sophisticated silicon transport mechanisms in plants. Such research may rationalize the creation of useful plant varieties based on their structures.

## Author contributions

YS and MS: organized and prepared the manuscript, and contributed to writing and reviewing the manuscript. All authors contributed to the article and approved the submitted version.

## Funding

This work was supported by JSPS KAKENHI grants JP16H06296, JP21H05034, JP22H04916 (MS), JP17H06879, JP19K16056, and JP21K15029 (YS) from MEXT, Japan.

## Conflict of interest

The authors declare that the research was conducted in the absence of any commercial or financial relationships that could be construed as a potential conflict of interest.

## Publisher’s note

All claims expressed in this article are solely those of the authors and do not necessarily represent those of their affiliated organizations, or those of the publisher, the editors and the reviewers. Any product that may be evaluated in this article, or claim that may be made by its manufacturer, is not guaranteed or endorsed by the publisher.
